# Distinct saliva DNA methylation profiles in relation to treatment outcome in youth with posttraumatic stress disorder

**DOI:** 10.1038/s41398-024-02892-1

**Published:** 2024-07-26

**Authors:** Judith B. M. Ensink, Peter Henneman, Andrea Venema, Jasper B. Zantvoord, Rosanne op den Kelder, Marcel M. A. M. Mannens, Ramón J. L. Lindauer

**Affiliations:** 1 Levvel, Academic Center for Child and Adolescent Psychiatry, Amsterdam, The Netherlands; 2 Department of Child and Adolescent Psychiatry, Amsterdam UMC, University of Amsterdam, Amsterdam Public Health, Amsterdam, The Netherlands; 3 Amsterdam UMC, Department of Human Genetics, Genome Diagnostics laboratory, Amsterdam Reproduction and Development Research Institute, Amsterdam, the Netherlands; 4 Amsterdam UMC, University of Amsterdam, Department of Psychiatry, Amsterdam Public Health, Amsterdam, the Netherlands; 5 Amsterdam UMC, University of Amsterdam, Department of Psychiatry, Amsterdam Neuroscience, Amsterdam, The Netherlands; 6 Research Institute of Child Development and Education, Amsterdam, The Netherlands, Amsterdam UMC, University of, Amsterdam, The Netherlands

## Abstract

In youth with posttraumatic stress disorder (PTSD) non-response rates after treatment are often high. Epigenetic mechanisms such as DNA methylation (DNAm) have previously been linked to PTSD pathogenesis, additionally DNAm may affect response to (psychological) therapies. Besides investigating the direct link between DNAm and treatment response, it might be helpful to investigate the link between DNAm and previously associated biological mechanisms with treatment outcome. Thereby gaining a deeper molecular understanding of how psychotherapy (reflecting a change in the environment) relates to epigenetic changes and the adaptability of individuals. To date, limited research is done in clinical samples and no studies have been conducted in youth. Therefore we conducted a study in a Dutch cohort of youth with and without PTSD (*n* = 87, age 8–18 years). We examined the cross-sectional and longitudinal changes of saliva-based genome-wide DNA methylation (DNAm) levels, and salivary cortisol secretion. The last might reflect possible abbreviations on the hypothalamic–pituitary– adrenal (HPA) axis. The HPA-axis is previously linked to DNAm and the development and recovery of PTSD. Youth were treated with 8 sessions of either Eye Movement Reprocessing Therapy (EMDR) or Trauma Focused Cognitive behavioral Therapy (TF-CBT). Our epigenome wide approach showed distinct methylation between treatment responders and non-responders on C18orf63 gene post-treatment. This genomic region is related to the PAX5 gene, involved in neurodevelopment and inflammation response. Additionally, our targeted approach indicated that there were longitudinal DNAm changes in successfully treated youth at the CRHR2 gene. Methylation at this gene was further correlated with cortisol secretion pre- and post-treatment. Awaiting replication, findings of this first study in youth point to molecular pathways involved in stress response and neuroplasticity to be associated with treatment response.

## Introduction

Posttraumatic stress disorder (PTSD) is a common mental health disorder observed in approximately 16% of youth exposed to traumatic events [1]. Youth with PTSD are troubled by frequent re-experiencing of the traumatic event, persistent avoidance, hyper arousal and negative alterations in cognition and mood [2]. If left untreated, these symptoms can interfere with social functioning and school performance, and have ongoing negative effects on the quality of life of the affected youth [3]. Furthermore, they are considered a crucial factor in shaping the vulnerability to depression and suicidality later in life [4]. This emphasizes the importance of effective treatments for youth with PTSD. Randomized controlled trials (RCTs) have demonstrated the efficacy of trauma-focused psychotherapies in youth with PTSD [5]. But response varies considerably among individuals, with high rates of heterogeneity in response and 20-50% of youth not benefiting sufficiently [6–9]. Several mechanisms have been associated with differential responses to treatment in youth, amongst them are biological factors, such as; epigenetic, endocrinological and neurological factors [10–12]. Besides the potential role of these biological factors as a predictor for treatment outcome, related studies have shown that symptomatic change is likely mediated by underlying biological mechanisms, such as epigenetic and endocrinological change [13]. DNA methylation (DNAm) is an important epigenetic mechanism which reflects epigenetic change and affects endocrine functioning [14]. DNAm represents the transcriptional status of a particular gene and can be influenced by both genetic and environmental factors [15, 16]. Provoked by early exposure to traumatic events, DNAm is assumed to be associated with altered hypothalamic-pituitary-adrenal (HPA) axis functioning, and it is related to the development and recovery of PTSD [17–19]. It is assumed that DNAm affects glucocorticoid functioning, in particular the release of stress hormones such as cortisol, the end product of HPA-axis activation, which is pivotal in several central mechanisms involved in PTSD, and trauma-focused treatment, such as the primary stress response, (emotional) memory consolidation, memory retrieval, reconsolidation and extinction learning [20–24]. In our prior study, we reported that higher pretreatment basal cortisol secretion was a potential indicator of treatment response in youth with PTSD [25]. Despite growing evidence showing that DNAm and endocrine mechanisms are important for a successful adaptation to stressful events, and play an important role in development and persistence of PTSD, translational studies in clinical practice are still rare, especially in youth. To the best of our knowledge only four studies [11–13, 26] have investigated changes in DNAm in relation to symptomatic response in adults with PTSD. The most recent study [13] examined the relation between DNAm and hydrocortisone treatment. This study identified epigenetic markers, previously linked to startle reaction and fear learning and memory processes, predicting both symptom change and PTSD recovery [11–13, 26]. Confirming the evidence from animal models showing that epigenetic changes can be dynamic and potentially reversible as a consequence of environmental programming [27, 28].

Objective and reliable predictive biological markers of treatment response and/or symptomatic change may have the potential to guide treatment selection and improve treatment efficacy. However, despite the promising results of the studies described above, there remains a considerable knowledge gap. First, it is important to recognize that translational studies in humans are still scarce, and so far have only been conducted in adults. These results cannot be automatically translated to youth, because other biological pathways and mechanisms are likely involved as both epigenetic and endocrinological regulation undergoes considerable developmental change [29–32]. Secondly, it remains insufficiently clear if changes in DNAm related to PTSD, adapt in reaction to trauma-focused psychotherapy, and how these treatment-related changes in DNAm are associated with longitudinal changes in the endocrine system, such as cortisol secretion. Third, the predictive value of baseline DNAm for treatment response is still insufficiently clear. Therefore, in this study we aim to address these knowledge gaps by investigating the cross-sectional and longitudinal changes in DNAm and cortisol secretion in relation to the treatment response of youth with and without PTSD. Youth with PTSD received trauma-focused psychotherapy. We used pre-and post-treatment exploratory methylome wide analysis (MWAS) and targeted analysis on differently methylated positions (DMPs) and regions (DMRs). In addition, we measured salivary cortisol to compare changes in cortisol secretion with findings from the methylation analysis.

## Results

### Participant characteristics and clinical outcomes

A summary of participant characteristics is shown in Table 1. Responders and non-responders did not differ in baseline sociodemographic, trauma, and clinical characteristics, apart from ethnicity and type of index trauma. At baseline 93.48% of participants met the full DSM-IV diagnostic criteria for PTSD, the remaining 6.52% met criteria for a partial PTSD diagnosis. The most common index trauma in the PTSD group was interpersonal violence, followed by sexual abuse. Youth with PTSD did not differ from trauma exposed controls (TEC) (*N* = *41*) in gender and age, but did differ at baseline in ethnicity, and type of index trauma. The average baseline CAPS-CA score in treatment responders was *M* = 51.14 points, *SD* = 23.0, and in non-responders *M* = 47.75 points, *SD* = 22.6 which is indicative of moderately severe PTSD. Post treatment mean total CAPS-CA scores improved at T2 (*M* = 32.51 points, *SD* = 23.04 *t*(46) = 9.67, *p* < .000), and T3 (*M* = 23.5 points, *SD* = 22.59 *t*(18) = 4.41, *p* < 0.000). See Table 1.

**Table 1:** Subject characteristics.

	Responders (*n* = 22)	Non-responders (*n* = 24)	Controls (*n* = 41)	*p*-value^a^
Sociodemographic characteristics				
Female (%)	40.9	67.7	53.7	0.215
Age (years; mean, SD)	12.09 (3.07)	12.92 (2.74)	11.70 (3.81)	0.379
West European Ethnicity (%)	73.7	47.8	85.4	0.006
Trauma characteristics				
Index trauma (%)				0.000*
Sexual abuse	22.7	20.8	0	
Interpersonal violence	36.4	50.0	19.5	
Accidents/Medical	13.6	12.5	61.0	
Other	27.3	16.7	19.5	
Repeated trauma exposure (%)	59.1	58.3	22.0	0.002*
Clinical characteristics				
CAPS-CA study entry T1 (mean, SD)^b^	51.14 (23.0)	47.75 (22.61)	-	0.170
CAPS-CA post treatment T2 (mean, SD)^b^	19.82 (16.14)	45.50 (21.48)	-	0.000*
CAPS-CA Follow-up treatment T3 (mean, SD)^b^	9.09 (7.80)	46.14 (19.1)	-	
Externalizing problem behavior T1 (%)^c^	53.3	20.0	26.7	0.037*
Internalizing problem behavior T1 (%)^c^	57.1	35.7	7.1	0.002*

*CAPS-CA* Clinician-Administered PTSD Scale for Children and Adolescents,

^a^*p*-values < 0.05 shown in bold. Independent samples t-test for continuous and x^2^ tests for categorical variables.

^b^Range: CAPS-CA total, 0–139,

^c^Internalizing and Externalizing behavior are reported if a child scored above clinical cut-off on the RCADS/ YSR and SDQ RCADS, Revised Child Anxiety and Depression Scale. *indicates a significant result.

### Longitudinal epigenetic trajectories related to treatment outcome in youth with PTSD

**Fig. 1:**
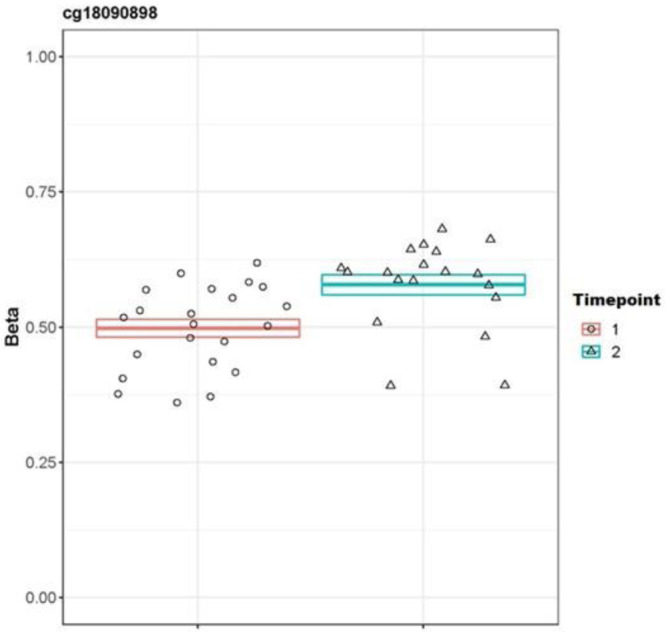
Treatment responders at the CRHR2 gene before and after treatment.

To examine the relation between successful treatment and DNAm we examined the effect of time of measurement (pre- (T1) and post-treatment (T2)) and follow-up treatment (T3) with the diagnosis of PTSD after treatment (responders versus non-responders). The results of the MWAS show no significant differences in treatment responders at T1 vs T2 (see Table 2a, b). Our targeted approach, showed one significant finding in treatment responders (T1 vs T2), at the *CRHR2* gene (*p* = 0.0003). Treatment responders showed increased methylation at T2, see Table 3, Fig. 1. A more in depth examination of this gene related to the glucocorticoid system, showed that increased methylation on the *CRHR2* gene in responders, was further associated with lower levels of basal cortisol at T2 (see Table 4). Additionally, our data indicates, however non-significant that in non-responders *CRHR2* methylation decreases from T1 to T2 (β = −0.013), this points to an opposite relation with cortisol secretion (decrease of methylation vs. increase of cortisol post-treatment), see Table 4. Furthermore *CRHR2* methylation is correlated with cluster D symptoms (hyperarrousal) before treatment, in non-responders (see Table 4). We didn’t detect any longitudinal differences between T1 and T3 in treatment responders. In treatment non-responders we didn’t detect any longitudinal differences between the T1 and T2, and T1 and T3.

**Table 2:** EWAS DMP and DMR analyses of treatment responders at T2 (pre-treatment) vs T2 (post-treatment).

**a: EWAS DMPs identified in the treatment responders at T2 compared with T1.**								
**Responders T1 VS T2**	**Gene**	**Probe**	**Chr**	**Position**	***m*-value**	**FDR**	**Log Beta**	**Delta Beta**
	*PHF15*	cg07525804	5	133914473	3,83E + 07	0.2293	1,21E + 09	0.0368
	*HMBS*	cg27472151	11	118956135	5,91E + 07	0.2293	1,63E + 09	0.0211
	*ARHGEF10*	cg14861020	8	1772352	1,03E + 08	0.2656	3,72E + 09	0.0258
		cg18890561	10	131988419	1,69E + 07	0.3276	8,21E + 09	0.0494
	*RAB27A*	cg24809382	15	55582033	3,33E + 08	0.3930	0.0002	0.0070

**b: EWAS DMRs identified in the treatment responders at T2 compared with T1.**								
**Responders T1 VS T2**	**Gene**	**Chr: start-end**	**Area**	**L**	**Cluster (L)**	***p*-value**	**FWER**	**Direction**
	*ARSG*	6: 291687-293285	0.557796234341063	7	7	3.15E-4	0.48	T2 > T1
	*SOX2OT*	17:6899207-6899577	0.540558928908835	8	20	3.13E-4	0.86	T2 > T1
	*BIN2*	10:12335523-123356041	0.514183280465409	7	14	3.98E-4	0.91	T2 > T1
	*PARVB*	3:1954890-195490033	0.490337023581752	7	9	4.49E-4	0.91	T2 > T1
	*LGR6*	17:80545175-80545434	0.446370934234037	5	6	5.34E-4	0.98	T2 > T1

Top 5 Responders vs. non responders at T2 (post-treatment). DMPs: Differently methylated positions Genome build (HG19), Gene: UCSC Reference Gene Name, chr: chromosome; m-value; FDR: false discovery rate adjusted *p*-value (Mval); LogBeta and DeltaBeta: delta differences between groups, based on average β-values.

Top 5 DMRs of association analyses of 1) Responders vs. non responders at T2 (post-treatment). *DMRs* Differently methylated regions Detected DMRs (L > 1) using Bumphunter; Genome build (HG19), Gene: UCSC Reference Gene Name, *chr:start-end* chromosome and position, *area* area bump, *L* Number of probes in DMR; cluster(L): number of probes in cluster; *FWER* Family-Wise Error Rate.

**Table 3:** Targeted DMPs analyses of treatment responders at T1 (pre-treatment) vs T2 (post-treatment).

**Responders T1 VS T2**	**Probe**	**Chr**	**Position**	***p*-value**	**DeltaBeta**	**Gene**	**Gene Feature**
	cg18090898	7	30739695	0.0003*	0.0802	*CRHR2*	1stExon
	cg17700633	19	13263997	0.0018	0.0187	*IER2*	5’UTR
	cg14849556	2	24991672	0.0052	0.0382	*NCOA1*	3’UTR
	cg08550353	6	1,34E + 08	0.0227	−0.0397	*SGK1*	TSS1500;Body
	cg26269677	5	76251688	0.0231	−0.0546	*CRHBP*	Body

Top 5 Targeted DMPs analyses of treatment responders at T1 (pre-treatment) vs T2 (post-treatment).DMPs: Differently methylated positions; chr: chromosome; *p*-value; DeltaBeta: delta differences between groups, based on average β-value: Genome build (HG19), Gene: UCSC Reference Gene Name; Gene feature: gene feature according Illumina manifest. Bonferonni corrected *P*-value 0.0003 *indicates a significant result.

**Table 4:** Correlations between cortisol secretion and CRHR2 methylation.

	cg18090898 (CRHR2) Total sample	cg18090898 (CRHR2) Responders	cg18090898 (CRHR2) Non-Responders
Pretreatment cortisol	−0.395^*^	−0.173	0.127
Posttreatment cortisol	−0.458*	−0.446*	0.439*
CAPS score Total T1	0.369^*^	0.471*	−0.122
Total score Cluster B T1	0.323	0.264	0.201
Total score Cluster C T1	0.088	0.222	−0.071
Total score Cluster D T1	0.446^*^	0.072	0.534*

*CAPS-CA* Clinician-Administered PTSD Scale for Children and Adolescents Pearson’s correlations between individual beta’s on selected DMP and cortisol secretion data in youth with PTSD and different clusters of PTSD symptoms and cortisol levels before and after trauma-focused psychotherapy. *Correlation is significant at the 0.05 level (2-tailed).

### Cross-sectional epigenetic differences and clinical outcomes in PTSD patients and TEC

Additionally, we performed a MWAS comparing the treatment responders, non-responders and trauma exposed controls (TEC) across the three different time points. Our DMP analysis revealed significant differences between responders and non-responders at T3, located on the *C18orf63* gene (cg15154763, FDR 0.05), indicating a decrease in *C18orf63* methylation in treatment responders (see Table 5). Our targeted approach yielded no additional significant findings. Despite not surviving our stringent multiple testing correction in our MWAS and targeted approach, of interest are our top 5 findings from our EWAS DMR analysis. These DMRs identified in the treatment responders vs non responders at T2 and T3 located at *RNF39, ALOX12, DUSP22, DIP2C* and *HOXA4, MUC4* genes, were all previously related to treatment outcome and/or development of PTSD, see Table 6 [11, 26, 33].

**Table 5:** EWAS DMPs identified in treatment responders vs. non responders at T2 (post-treatment), and T3 (follow-up).

**Responders vs Non Responders T2**	**Probe**	**Chr**	**Position**	**FDR**	**DeltaBeta**	**Gene**	**Gene Feature**
	cg24809382	15	55582033	0.99	0.0076	RAB27A	TSS1500
	cg17251423	5	139088815	0.99	−0.1560	CTB-35F21.1	Body
	cg08273874	16	1060765	0.99	0.0554		-
	cg00313642	3	196014711	0.99	0.0414	PCYT1A	Body
	cg08313638	10	12110577	0.99	−0.0443	DHTKD1	body
**Responders vs Non Responders T3**	cg15154763	18	71982463	0.05**	0.2259	C18orf63	TSS1500
	cg11198041	14	35344866	0.83	0.0801	BAZ1A	TSS200
	cg17700633	19	13263997	0.83	−0.0246	IER2	5’UTR
	cg07275860	12	120554537	0.83	0.0521	RAB35	5’UTR
	cg14362428	6	31116408	0.83	−0.0751	CCHCR1	Body

Top 5: EWAS DMPs from our cross sectional analysis between treatment responders vs. non responders at T2 (post-treatment), and T3 (follow-up). DMPs: Differently methylated positions. Genome build (HG19 chr: chromosome; FDR: false discovery rate based on bacon adjusted *p*-value (Mval); DeltaBeta: delta differences between groups, based on average β-value; Gene: UCSC Reference Gene Name; Gene feature: gene feature according Illumina manifest **indicates a epi-genome wide significant result.

**Table 6:** EWAS DMRs identified in treatment responders vs. non responders at T2 (post-treatment), and T3 (follow-up).

**Responders vs Non Responders T2**	**Chr**	**Start**	**End**	**L**	**Cluster (L)**	**FWER**	**Direction**	**Gene**
	6	30039380	30039801	10	36	0.39	R > NR	*RNF39*
	6	291687	293285	10	10	0.81	R > NR	DUSP22
	17	6899207	6899577	9	23	0.87	NR-R	ALOX12
	10	12335523	123356041	6	23	0.92	R > NR	*FGFR2*
	3	19548900	195490033	6	11	0.93	NR-R	*MUC4*
**Responders vs Non Responders T3**	6	291687	293285	10	10	0.51	R > NR	*DUSP22*
	10	530635	532357	15	21	0.55	R > NR	DIP2C
	7	2717021	27171051	14	32	0.56	NR-R	HOXA4
	3	1954890	195490033	11	11	0.89	NR-R	*MUC4*
	12	47219626	47219920	8	13	0.93	R > NR	*SLC38A4*

Top 5 EWAS DMRs from our cross sectional analysis between treatment responders vs. non responders at T2 (post-treatment), and T3 (follow-up). DMRs: Differently methylated regions; Detected DMRs (L > 1) using Bumphunter; chr start-end: chromosome and position; L: number of probes in DMR; cluster(L): number of probes in cluster, *FWER* Family-Wise Error Rate Direction: *R* Responders, *NR* Non responders.

To control for the effect of trauma exposure and time, we performed an additional analysis between the responders [22] and non-responders [24] and the TEC. We detected no significant DMR’s or DMP’s between the clinical group and the controls at T1 and T2. At T3 we detected one significant finding between the clinical groups and the control group at one DMR located on chr2: *NUP35* (FWER 0.05) Clinical groups showed hypermethylation compared to controls.

## Discussion

To the best of our knowledge, this is the first study to detect DNAm in youth with PTSD in relation to trauma-focused psychotherapy response.

Our epigenome-wide *cross-sectional analysis* showed significant differences between responders and non-responders at T3 (follow-up), located on the *C18orf63* gene. Indicating a decrease in *C18orf63* methylation in treatment responders. A prior epigenome-wide methylation study found a relationship between *C18orf63* methylation and socioeconomic status (SES) in placentas from preterm infants [34]. We consider these findings relevant to our findings since variance in SES is associated with many health outcomes. Including the development of neurodevelopmental and neurobehavioral disorders in children, as well as poorer adult health status and shorter life expectancy [34–36]. The association with DNAm might imply biological embedding of SES adversity within critical developmental periods, which in turn could affect long-term child health outcomes [34]. On a molecular level *C18orf63* is related to the PAX5 gene (*C18orf63* Gene - GeneCards | CR063 Protein | CR063 Antibody). *PAX5* was previously found to be related to PTSD and depression [37, 38]. Thompson and colleagues suggested that the perceived capacity of *PAX* genes to respond to stress is relative high and PAX genes seem to respond within the central nervous system (CNS) as well as interact with a damaged or regenerating environment [39]. In addition the *PAX5* gene plays an important role in inflammatory responses (by regulating B-cell differentiation). Multiple studies observed the associated between elevated levels of inflammation in PTSD [40, 41]. Additionally, DNA methylation in genes related to the inflammatory responses are observed as well in PTSD [42–44]. These findings indicate that alterations of specific peripheral inflammatory markers maybe related (or induced by) alterations on specific DNAm sites. In addition to these outcomes our MWAS DMR analysis showed a subsequent amount (nominal significant) of overlap with DMR’s reported in the first treatment related studies in adults with PTSD, annotated to *RNF39*, *DUSP22*, *DIP2C* and *HOXA4*, *MUC4* genes [11, 13, 26, 45]. We detected one locus of interest regarding responsivity to treatment located on the *ALOX12* genomic region. *ALOX12* is the predominant LOX enzyme in the brain and previously this location was related to cortical thickness in PTSD, responsivity to oxidative stress en elevated inflammatory responses [46–48]. Interestingly, the few structural neuroimaging studies of trauma-focused psychotherapy in adults that found evidence for pre-to post treatment changes within several regions of the cortex, including the prefrontal cortex, cingulate cortex and insula [46, 49–51]. However, imaging data from the cohort used in this study did not replicate the relationship between treatment response and prefrontal cortex volume change, yet we did show that non-response to trauma-focused psychotherapy was characterized by longitudinal bilateral volume decrease in both the posterior and anterior insula [52]. The results from our longitudinal analysis in treatment responders and non-responders did not indicate any significant effects that survived our multiple testing correction. Only at T3 we detected one significant finding between the clinical groups and the control group at one DMR annotated to the *NUP35* gene. Clinical groups showed hypermethylation compared to controls. Differential methylation on *NUP35* was mentioned before in relation to variation in cognitive function between twins [53].

However, our targeted approach showed interesting *longitudinal* outcomes. We observed differential DNAm before and after treatment in treatment responders at the *CRHR2* genomic region (cg18090898). In response to acute stress, the hypothalamic corticotrophin releasing hormone (CRH) interacts with CRHR2 (a corticotrophin releasing hormone receptor), and it stimulates the anterior pituitary to release adrenocorticotropic hormone. In turn this hormone stimulates the adrenal cortex to release glucocorticoid hormone (cortisol) [54, 55]. Interestingly, in our sample substantial correlations between DNAm at this site and cortisol secretion before and after treatment were observed. Increased methylation on *CRHR2* genomic region in treatment responders was associated with an observed increase of cortisol during trauma-related psychotherapy. An observed increase of cortisol during trauma-related psychotherapy, as shown in our sample in treatment responders, may enhance the consolidation of the memories of fear and other strong emotions to facilitate coping with re-exposure [56].

The direction of effect of these findings seems in line with the results of previous studies. These studies showed that lower levels of pretreatment cortisol and lower cortisol change during treatment are related to poorer treatment outcome [25, 57]. Despite that we could not confirm other previous DNAm findings in relation to treatment response previously reported in specific glucocorticoid related regions, such as differential methylation on the *FKBP5* and *NR3C1* genes. Our findings on the *CRHR2* genomic region do support the hypothesis that DNAm at stress-related genes are related with glucocorticoid signaling and with trauma-focused psychotherapy response. This in turn could be possibly related to emotional reactivity during treatment, previously identified as a risk factor for ongoing disbalance after exposure to traumatic events early in life [32]. Only limited information is available on the relation with gene expression within this area. Mainly because of non-matching tissues in common used gene expression databases and the absence of information about mQTLs. However, since cg18090898 is located on first exon of the gene, in general it can be assumed that hypermethylation (similar as to gene promotor hypermethylation) is related to less gene expression [58].

This study has several strengths and limitations. The major strengths of our study are the longitudinal design and standardized PTSD and biological assessments in a unique sample of youth with PTSD, before and after treatment. Furthermore, we used an unbiased methylome-wide approach, extended with a literature based targeted approach, and we included a trauma exposed reference group to control for effect of trauma. We used stringent exclusion criteria and a strict multiple testing correction. However there are also several limitations that have to be acknowledged. Firstly, the relative small size of the groups included in this study, limited us to detect significant epigenome wide results [59]. This also limited us in our ability to examine differences between treatment responders and non-responders for both treatment conditions separately. Despite that both are equally effective trauma-focused psychotherapies in youth [60]. And both therapies share multiple common elements such as exposure to and reprocessing of traumatic memories [61], we acknowledge that there is need for additional research. Information from cohorts with larger sample-sizes, or combined meta-analyses might provide a deeper insight between possible differential effects of both therapies in relation to epigenetic predictors of treatment response and/or biological changes during treatment. Furthermore, the considerable drop-out rate of randomized patients lost at follow-up, restricted us further in our longitudinal analysis. Although, dropout rates in our study reflect routine clinical practice, there is a possibility that drop-out could have influenced our main findings through attrition bias.

Secondly, there are several limitations regarding our post-hoc cortisol measures, such as the reaction on our script driven imagery procedure and the lack of an additional circadian cortisol secretion measure. These are described in more detail in our previous paper [25]. Furthermore, despite that we’ve tried to account for most confounding factors, we weren’t able to include all known factors to influence endocrine function, for example we omitted inquiry on menstrual and pubertal stage. Lastly, another possible limitation is the relevance of methylation in saliva to other tissues such as the brain, given that methylation differences across tissues are substantial. Despite that consistent effects of various methylation quantitative trait loci (mQTLs) are found across tissues [62], partial evidence exists on cross-tissue consistent findings [63]. Therefore a cautious approach to the interpretation of findings obtained from single tissue analyses is needed. In future studies we suggest to link PTSD and treatment outcome and DNAm with specific brain and endocrine related endophenotypes. Given the expected age and time dependent differences, especially considering HPA-axis and brain plasticity, during critical periods it would be helpful to increase sample sizes and include youth already early in life thereby increasing feasibility to differentiate across the developmental stages. In addition the use of continuous outcomes (using symptom dimensions scores instead of a dichotomous diagnosis) reflecting the different symptoms of PTSD and associated emotional and behavioural problems might be considered in future research.

## Conclusion

In conclusion, this is the first study in youth with PTSD, that shows the association between successful trauma-focused psychotherapy with specific DNAm changes Overall our results do support and extend previous outcomes presenting DNAm change in relation to treatment response in youth with PTSD, presenting DNAm change in specific genes related to exposure to trauma, the development of PTSD and its related psychological treatments [11, 12, 64]. The study provides further insight in underlying biological mechanisms, and indicates how biological mechanisms might interact with symptomatic change in youth with PTSD. Our results provide novel insights that may contribute to the discovery of the epigenetic mechanisms underlying a successful treatment of PTSD, especially related to HPA-axis related endophenotypes. We expect that the findings may help to better understand how psychological and biological systems interact in order to improve and individualize treatment outcomes, since ideally we aim to prevent ongoing PTSD symptoms in youth and its severe consequences by improving interventions, that are better tailored to each individual patient.

## Method

### Cohort and study design

In the present study a MWAS, targeted epigenetic approach, cortisol and clinical assessments were performed in a cohort of youth (*N* = 87, 54.1% female, aged 8-18), Youth with PTSD (*N* = 46) were matched for age and sex with a control group of trauma exposed controls (TEC) without PTSD (*N* = 41). Youth with PTSD were recruited between April 2011 and September 2018 at the outpatient child psycho-trauma center of the department of child and adolescent psychiatry, de Bascule in Amsterdam, The Netherlands. The participants were part of a larger RCT comparing trauma-focused cognitive behavioural therapy (TF-CBT) and eye movement desensitization and reprocessing (EMDR) [65]. They were referred by child welfare services, physicians or their general practitioner. TEC were recruited between June 2011 and September 2018 through local elementary- and high schools by researchers JBZ, RodK and JBME. Exclusion criteria for both groups included imminent suicidality, history of psychotic disorder, substance abuse or dependence; IQ < 70; unstable medical condition; recent use of psychotropic medication (past 4 weeks; 6 weeks for fluoxetine); and possibility of pregnancy in females. All participants received a monetary incentive for participation (€5 for each assessment). In both groups written parental and youth assent were obtained for all participants. All procedures were approved by the Medical Ethical Committee of the University Medical Center.

### Procedures and measures

Diagnosis for PTSD in the clinical group were established clinically by an experienced child and adolescent psychiatrist or psychologists according to the DSM-IV-TR criteria using both child reports on the Clinician-Administered PTSD Scale for Children and Adolescents (CAPS-CA), which is a reliable semi-structured interview [66]. In addition caregiver information was obtained from the PTSD scale of the Anxiety Disorders Interview Schedule – Parent Version (ADIS-P) [67]. (Partial) PTSD diagnosis was determined using joint-child and caregiver reports on individual symptoms. A symptom was established as present, if either child or caregiver reported its presence. All participants were required to have a CAPS-CA total score indicating at least mild PTSD symptom severity ( > 20 points). Clinical evaluations were performed pre-treatment (T1), post-treatment (8 sessions of psychotherapy) and follow-up (6 months post-treatment). Based on the psychometric properties of the CAPS (-CA) and previous treatment outcome studies using the CAPS-CA, we used ≥ 30% reduction of CAPS-CA total score as response criterion for clinically meaningful improvement [68, 69]. In the TEC exposure to traumatic events were validated according to A1 and A2 criteria of DSM-IV-TR (American Psychiatric Association, 2000) using the Children’s Revised Impact of Event Scale (CRIES) [70, 71]. Information about additional internalizing and externalizing symptoms were measured using youth and caregiver reports, with the Revised Children’s Anxiety and Depression Scale (RCADS) and the Child Behavioral Checklist (CBCL) and Youth Self Report (YSR) [72–76]. Clinical characteristics of both groups are shown in Table 1.

### Treatment

After study entry all patients were randomized to receive either protocolled sessions of trauma-focused cognitive behavioral therapy (TF-CBT) or eye movement desensitization and reprocessing (EMDR) by trained and experienced therapists. Supervision by experts on TF-CBT and EMDR was provided throughout the study. Treatment protocols, training and supervision of therapists, as well as treatment fidelity have been described in detail previously [25, 65].

### DNA methylation

Three milliliters of saliva were collected and stored in Oragene DNA sample collection kits (DNA Genotek, Canada). DNA was extracted using a Gentra autopure LS system following manufacturers protocol. Genomic DNA samples were resolved on a 1% agarose gel to verify that the DNA was of high molecular integrity. Quantification of the DNA was determined using Qubit (Qiagen, USA). Five hundred nanograms of genomic DNA was sodium bisulfite–treated for unmethylated cytosine (C) to thymine (T) conversion using the EZ DNA Methylation-Gold kit (Zymo Research). Prior to DNA methylation profiling, cases and controls were randomized across the 96 well plates. Technical replicates (*n* = 8) were included for quality control of array, monitoring potential batch effects. Briefly, converted DNA was amplified, fragmented, hybridized, and scanned using the Illumina Methylation EPIC 850k Beadchip, following the manufacturer’s guidelines.

### Cortisol

Information about SDI and cortisol collection is published previously [25]. In short, all participants performed a standardized protocol for script driven imagery (SDI) [77], in combination with the collection of five samples, 10 min and 1 min prior to trauma script imagery as well as 10 min, 20 min, and 30 min after trauma script imagery. Next, For each participant we determined if SDI induced a cortisol stress response [78]. This was defined as an increase of at least 1.5 nmol/l compared to baseline levels. Area under the curve with respect to ground (AUCg) and increase (AUCi) were derived using the trapezoidal formulas [79]. As the SDI failed to induce a cortisol stress response in all but two participants, we did not use the AUCi as measure of cortisol responsivity in further analyses. Therefore, the AUCg was used as a measure of total cortisol output which captures basal cortisol secretion as opposed to stressor reactivity. AUCg was examined for normality of distribution within each group. Non normally distributed data were log-transformed. See also for more detailed information our previous paper for additional information about the data handling [25].

### Statistics

The distribution of baseline clinical, trauma and demographic characteristics across responders, non-responders and TEC was examined using X^*2*^-tests for categorical variables, independent sample *t*-tests for normally distributed continuous variables and Mann-Whitney tests for non-normally distributed continuous variables. Paired sample *t*-test were used to examine pre- to post-treatment symptom change. These statistical analyses were performed using SPSS version 24.0 (SPSS Inc., Chicago IL, USA).

Raw DNA methylation profiles were imported into the statistical programming environment R (v.3.4.2) using the Bioconductor (v3.13) and the minfi (v1.38.0) package [41]. Data quality control was performed using MethylAid. We used the following MethylAid thresholds for quality control; 10.5 for methylated and unmethylated intensities, 12 for overall quality control, 11.75 for bisulphite control, 12.75 for hybridization control, and a detection *P*-value of 0.95(v1.26.0) [42]. At this stage we removed one sample, because it did not meet our quality control criteria (Follow-up measurement). Further statistical analysis were based on M-values calculated as log2 (beta/(1-beta). Probe expressing infinite values were removed from the dataset. Visualization of associations were based beta-values, i.e. methylation index, which ranges between 0% (methylated) and 100% (methylated). In the next step principal component analysis (PCA) was applied in order to detect any outliers and to evaluate concordant sexes of samples. Moreover, we performed hierarchical clustering analysis using 11 single nucleotide polymorphisms, known to be present on the Illumina EPIC array, to evaluate the concordance of the genotype within the longitudinal sample sets. Next we normalized the dataset applying the function *funnorm* and we removed all probes annotated to the allosomes, susceptible for cross-hybridization, and probes known to confounded by genetic variation with a minor allele frequency >1%.

Subsequently we correlated meta-data, i.e. sex, age and technical potential confounders (slide and array position) with the first eight principal components. These first eight principal components explained together most of the variance. The qq-plots (of the expected p values versus the observed p values) had a lambda of >.0.85, <1.15 this indicated absence of type-I error inflation and no artificial differences between groups. From this analysis we defined the following statistical models for paired and unpaired analyses respectively:

$${Paired\; analysis}:{methylation} \sim {group}$$

$${Unpaired\; analyses}:{methylation} \sim {group}+{sex}+{age}$$

Differential methylated positions (DMPs) were obtained using *limma*. For the detection of DMRs we applied the *bumphunter* function, wherein we used delta beta difference thresholds of 10% and 5% for paired and unpaired analyses respectively. We analyzed the main (DMPs and DMRs) effects longitudinal and crossectional between the following groups: (1) PTSD vs TC on the three different time points; T1: baseline level, before treatment, T2: directly after 8 sessions of trauma-focused treatment, T3: follow-up 6 months after treatment. We assumed a false discovery rate (FDR) < .05 for DMPs and a familywise error rate (FWER) of 0.05 for DMRs as significant. For our targeted approach we selected a limited number of replication loci (N_DMP_ = 170, N_DMR_ = 30), which were based on a literature search which included published MWAS data in youth with PTSD, an previous published treatment studies until June 2021. We also added several candidate loci associated with glucocorticoid functioning and previously related to PTSD development. See supplementary Table 1. In our targeted approach we applied Bonferroni correction threshold wherein we assumed a *p* < 0.002 significant for the DMR approach, and a *p* < 0.0003 significant for the DMP approach. The Bonferroni threshold was the product of dividing critical α = 0.05 by the number of DMR’s and DMPs of interest extracted from previous studies. In total we tested 170 DMP’s and 30 DMR’s, which we considered relevant based on previous studies.

Next, we calculated Pearson’s correlation in SPSS between DNAm and our cortisol data. We measured the relation between DNA methylation at significant DMP’s either derived from our epigenome-wide or our targeted epigenetic analyses (if considered relevant to the glucocorticoid system) and cortisol secretion.

### Supplementary information

sTable1
