# Supplementary material for: Distinct saliva DNA methylation profiles in relation to treatment outcome in youth with posttraumatic stress disorder
Source: Transl Psychiatry. 2024 Jul 26;14:309. doi: 10.1038/s41398-024-02892-1 (PMC11282249; doi:10.1038/s41398-024-02892-1)
Supplement: Supplementary file 1 — sTable1 [file 41398_2024_2892_MOESM1_ESM.docx]

**STable 1**: Selected DMPs and DMRs for targeted approach

| cg17700633 cg21972431 cg26196496 cg00862770 cg03591753 cg06937024 cg07485685 cg07843056 cg11845071 cg15929276 cg19226017 cg25114611 cg03546163 cg06087101 cg07485685 cg08423118 cg16052510 cg16586394 cg17860381 cg19645279 cg20728768 cg21702128 cg25579735 cg01170198 cg16562342 cg20012601 cg05616442 cg00130530 cg00629244 cg01277438 cg01294490 cg01312837 cg01967637 cg02521996 cg02564102 cg02665568 cg02842899 cg03857453 cg03883275 cg04444450 cg04444450 cg05121010 cg05790989 cg05790989 cg06521673 cg06613263 cg06937024 cg07061368 cg07528216 cg07733851 cg08586216 cg08845721 cg09566021 cg10300814 cg10847032 cg10913456 cg10913456 cg11152298 cg11321922 cg11540119 cg11916669 cg13103915 cg13135255 cg13344434 cg13648501 cg13986355 cg14284211 cg14558428 cg14642437 cg14825287 cg14849556 cg15115787 cg15910486 cg15912732 cg16005389 cg16012111 cg16012111 cg16182267 cg16224829 cg16335926 cg16569373 cg17030679 cg17085721 cg17406386 cg17617527 cg18019515 cg18068240 cg18071894 cg18146873 cg18484679 cg18849621 cg19014730 cg19261497 cg19457823 cg20090430 cg20509117 cg20509117 cg20730067 cg20813374 cg21979215 cg22237988 cg23273257 cg23416081 cg23462257 cg23523922 cg23624957 cg23751680 cg24026230 cg24295963 cg24307117 cg25368824 cg25535999 cg26049684 cg26464411 cg26495008 cg26560981 cg27345592 cg22046703 cg24738082 cg01049782 cg01819552 cg04923928 cg23185751 cg03667083 cg09516959 cg04922810 cg07658503 cg21773872 cg01972879 cg13094036 cg02712145 cg01718447 cg15615793 cg01972879 cg23185751 cg18090898 cg26269677 cg05620787 cg13777717 cg17238830 cg26196496 cg16545105 cg21199406 cg01071966 cg21842274 cg17448335 cg05183646 cg05966641 cg25661219 cg03146155 cg08550353 cg21834463 cg03762694 cg21676440 cg14905466 cg21078322 cg09404376 cg08647910 cg13307058 cg07340870 cg25025235 cg11856561 cg06642177 cg25150212 cg12871835 cg17284168 cg12009778 cg24688636 cg02904344 cg21064939 |
| --- |

*Note:* Selected DMPs (Differentially methylated positions)

| **Name** | **loc** | **Start** | **End** |
| --- | --- | --- | --- |
| TNXB | chr 6 | 32064573 | 32064660 |
| PM20D1 | chr 1 | 205818956 | 205819609 |
| TNXB | chr6 | 32063901 | 32064258 |
| DUSP22 | chr6 | 291687 | 293285 |
| GDF7 | chr2 | 20870087 | 20871401 |
| SLC1A4 | chr2 | 65217211 | 65217623 |
| KLHL35 | chr11 | 75139390 | 75139680 |
| ZNF714 | chr19 | 21264896 | 21265421 |
| OLFM3 | chr1 | 102312608 | 102312671 |
| NR3C1_1 | chr5 | 142782046 | 142782472 |
| NR3C1_2 | chr5 | 142783585 | 142783906 |
| NR3C1_3 | chr5 | 142784559 | 142784950 |
| SLC6A4_1 | chr 17 | 28562939 | 28563283 |
| SLC6A4_2 | chr 17 | 28562574 | 28562952 |
| SLC6A4_3 | chr 17 | 28562328 | 28562682 |
| OXTR-1 | chr 3 | 8799262 | 8799615 |
| OXTR-2 | chr 3 | 8800371 | 8800739 |
| FKBP5 | chr 6 | 35.558.322 | 35558593 |
| MUC4 | chr3 | 195489306 | 195490309 |
| APOB | chr2 | 21266500 | 21267212 |
| EDN2 | chr1 | 41950237 | 41950392 |
| ZFP57 | chr6 | 29648271 | 29648623 |
| GPX6 | chr6 | 28478268 | 28478579 |
| CFAP45 | chr1 | 159869902 | 159870134 |
| AFF3 | chr 2 | 100720526 | 100720529 |
| TP73 | chr 1 | 3600735 | 3600879 |
| UBCLP1 | chr 5 | 158689508 | 158689629 |
| RPL13P | chr 6 | 28829171 | 28829433 |
| DMR11_BOKS | chr 19 | 11784955 | 11785188 |
| DMR12_BOKS | chr 17 | 6558365 | 6558440 |

*Note:* Selected DMRs (Differentially methylated positions)
